# Late-Onset Moyamoya Disease Presenting With Acute Hemiparesis in a 77-Year-Old Patient: A Case Report

**DOI:** 10.7759/cureus.108884

**Published:** 2026-05-15

**Authors:** Hassan Waheed, Ariel Litinski, Vismay Patel, Mohammed Mirza, Nathan Farkas

**Affiliations:** 1 Internal Medicine, Hudson Regional Health, Bayonne, USA; 2 Academy for Biotechnology, Morris County School of Technology, Denville, USA; 3 Neurology, Hudson Regional Health, Bayonne, USA

**Keywords:** ais (acute ischemic stroke), collateral vessels, digital subtraction angiography(dsa), elderly patients, intracranial arterial stenosis, left-sided hemiparesis, moyamoya disease (mmd), moyamoya vasculopathy, pontine infarction, suzuki staging

## Abstract

Moyamoya is a progressive cerebrovascular condition leading to narrowing of the distal internal carotid artery (ICA) and its branches with subsequent development of abnormal collateral vessels around the ischemic brain area. Moyamoya disease is mostly encountered in children and young adults. It is rare in older patients with acute ischemic stroke and requires maintaining a broad differential diagnosis despite the presence of a rare cerebrovascular condition.

A 77-year-old woman presented with left-sided weakness outside the thrombolysis window. Imaging revealed moyamoya vasculopathy and an acute right pontine infarct likely embolic in origin, while echocardiography showed an intra-atrial shunt. She was managed with dual antiplatelet therapy and supportive care before discharge to rehabilitation.

Moyamoya disease in elderly patients is very rare and may represent a slowly progressing, long-standing process, where opportunities to diagnose it at an earlier age were missed. Secondary causes of moyamoya, including autoimmune disease and atherosclerotic arteriopathy, which is more prevalent in elderly patients, have to be considered. Appropriate choice of imaging modalities in acute ischemic stroke may help to narrow the differential diagnosis and influence further therapeutic approaches.

## Introduction

Moyamoya disease is a rare cerebrovascular condition associated with progressive stenosis of the distal internal carotid artery (ICA) and its branches, the anterior cerebral artery (ACA) and the middle cerebral artery (MCA). It is characterized by the subsequent development of abnormal vascular collaterals that are prone to hemorrhages [1]. Thus, it frequently presents as an acute ischemic or hemorrhagic stroke [2].

Moyamoya disease has a bimodal age distribution, being most encountered in children and young adults [3]. While most prevalent in East Asian nations, such as Japan, China, and Korea, other ethnic groups are affected as well [4,5]. In the United States, the incidence of moyamoya arteriopathy is 0.57 per 100,000, and the average age is 31.6 years [6]. The female population is affected approximately twice as often as males [5].

The pathogenesis of moyamoya disease involves genetic and epigenetic factors. Familial cases of moyamoya have been reported; however, cases in which only one of two identical twins was affected have also been described, suggesting a multifactorial etiology [7].

Moyamoya vasculopathy can be seen in other acquired and congenital conditions. When associated with these comorbidities, moyamoya is referred to as moyamoya syndrome as opposed to idiopathic moyamoya disease [8]. Of note, atherosclerosis can be associated with moyamoya arteriopathy as well [8]. The gold standard diagnostic modality for moyamoya is cerebral digital subtraction angiography (DSA); however, brain MRI and CT angiograms may exhibit suggestive radiographic signs. Treatment of moyamoya involves surgical revascularization with or without concomitant medical therapy, including antiplatelet agents [8].

Moyamoya disease is a rare cause of ischemic stroke in elderly patients. The oldest reported patient with moyamoya disease was 82 years old [9]. Even when present, alternative etiologies, such as thromboembolic and atherosclerotic vascular disease, should be considered in the differential diagnosis of acute neurological deterioration. Thromboembolic stroke is typically characterized by an abrupt onset and infarcts in multiple vascular territories, whereas atherosclerotic disease more commonly presents with progressive stenosis and territorial infarcts corresponding to affected vessels [10].

This case is presented to illustrate the diagnostic complexity that arises when moyamoya vasculopathy is encountered in an elderly patient and to demonstrate how careful correlation of multimodal imaging findings can guide appropriate stroke mechanism attribution and secondary prevention.

## Case presentation

A 77-year-old Filipino woman presented in March 2026 to the emergency room with a new onset of left-sided weakness of both upper and lower extremities. She had no additional complaints. Her past medical history was significant for diabetes mellitus type 2, hypertension, a history of one transient ischemic attack, scalp psoriasis, and seizure disorder. Her medication list included metformin, losartan, and glimepiride. She received a secukinumab subcutaneous injection a week ago for mild plaque psoriasis. Her family history was unremarkable. She did not have any allergies, was a lifelong non-smoker, and did not drink alcohol or use recreational drugs.

Her vital signs were as follows: blood pressure, 148/90 mm Hg; heart rate, 81 bpm; regular, respiratory rate, 18/min; oxygen saturation 98% on room air. Her blood glucose level was 292 mg/dL. Neurological examination revealed 2/5 motor strength in the left distal and proximal upper and lower extremities, with no other focal deficit. The remainder of the physical examination, including the skin examination, was unremarkable. The initial NIH Stroke Scale (NIHSS) score was 5, consistent with a mild-to-moderate neurological deficit [11]. Laboratory values are described in Table 1.

**Table 1:** Admission laboratory values BUN: blood urea nitrogen; AST: aspartate aminotransferase; ALT: alanine aminotransferase; TSH: thyroid-stimulating hormone; HbA1C: hemoglobin A1c; LDL: low-density lipoprotein; HDL: high-density lipoprotein.

Test	Result	Reference Range
Hemoglobin	14.3 g/dL	12.0–16.0 g/dL
Hematocrit	42.8 %	36–46 %
Platelets	210 ×10³/µL	150–400 ×10³/µL
BUN	26 mg/dL	7–20 mg/dL
Creatinine	0.9 mg/dL	0.6–1.3 mg/dL
AST	29 U/L	10–40 U/L
ALT	35 U/L	7–56 U/L
Sodium (Na)	139 mmol/L	135–145 mmol/L
Potassium (K)	3.9 mmol/L	3.5–5.0 mmol/L
Glucose	292 mg/dL	70–99 mg/dL
Troponin I	<0.01 ng/mL	<0.04 ng/mL
TSH	1.02 µIU/mL	0.4–4.0 µIU/mL
Folate	15.2 ng/mL	2–20 ng/mL
Homocysteine	11 µmol/L	5–15 µmol/L
Vitamin B12	370 pg/mL	200–900 pg/mL
HbA1C	8.7 %	<5.7 %
Triglycerides	176 mg/dL	<150 mg/dL
Cholesterol	201 mg/dL	<200 mg/dL
LDL	94 mg/dL	<100 mg/dL
HDL	64 mg/dL	>40 mg/dL

**Figure 1:**
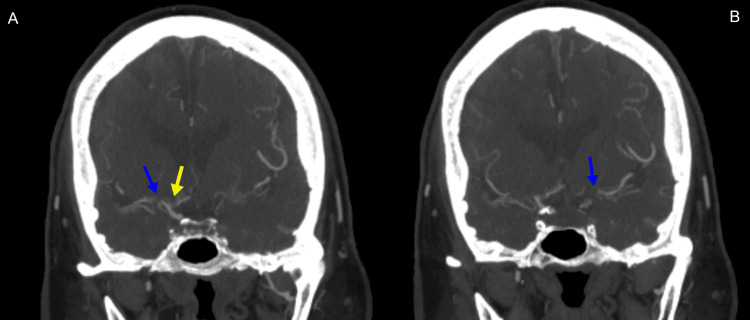
CTA demonstrating bilateral steno-occlusive lesions. CTA demonstrating bilateral steno-occlusive lesions involving the bilateral proximal M1 (blue arrow) and A1 segment (yellow arrow). Panel A demonstrates right-sided pathology, while Panel B demonstrates left-sided pathology. CTA: computed tomography angiography

A non-contrast CT head demonstrated no hyperdense intracranial hemorrhage or acute hypodense infarct. Since the patient was last seen without neurological deficit more than 12 hours ago, no systemic thrombolysis was considered. CTA of the head and neck was performed. Findings were consistent with moyamoya disease, including prominent lenticulostriate arteries, representing collateral vessels (Figure 1).

**Figure 2:**
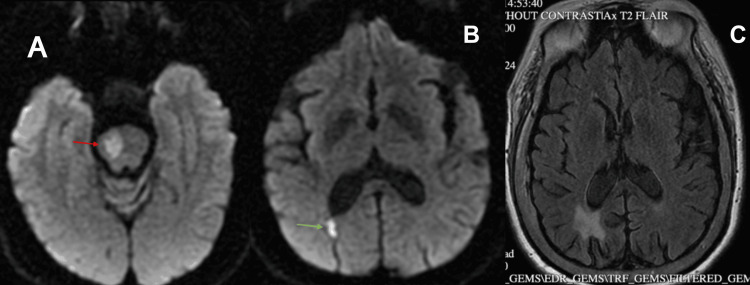
MRI showing areas of acute stroke A: Brain MRI, DWI demonstrating acute infarct in the right paramedian pons (red arrow). B: Brain MRI, DWI demonstrating posterior parietal periventricular infarct (green arrow). C: FLAIR imaging demonstrating periventricular white matter changes. DWI, diffusion-weighted imaging; FLAIR, fluid-attenuated inversion recovery

A subsequent brain MRI (Figure 2) demonstrated diffusion restriction on diffusion-weighted imaging (DWI) with a corresponding low apparent diffusion coefficient (ADC) signal, consistent with an acute right pontine infarct within the basilar perforator territory. An additional hyperintense signal was noted in the posterior parietal periventricular white matter, most consistent with an embolic etiology. The patient was transferred to the ICU for acute ischemic stroke and managed with permissive hypertension, maintaining euvolemia and normothermia. After speech and swallow evaluation, the patient was started on dual antiplatelet therapy and high-dose statin therapy, including aspirin 81 mg daily, clopidogrel 75 mg daily, and atorvastatin 80 mg daily.

**Figure 3:**
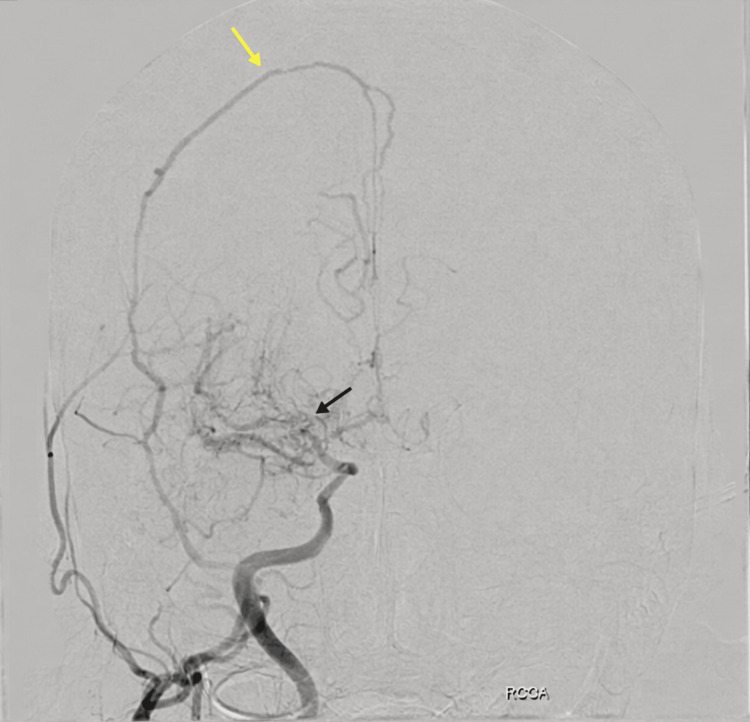
DSA showing moyamoya vasculopathy. RCCA angiogram demonstrating severe proximal M1 and A1 stenosis. The black arrow indicates prominent lenticulostriate collateral vessels. The yellow arrow indicates collateral flow from the right MMA frontal branch to the right anterior cerebral artery ACA. DSA: digital subtraction angiography; RCCA: right common carotid artery; MMA: middle meningeal artery; ACA: anterior cerebral artery

**Figure 4:**
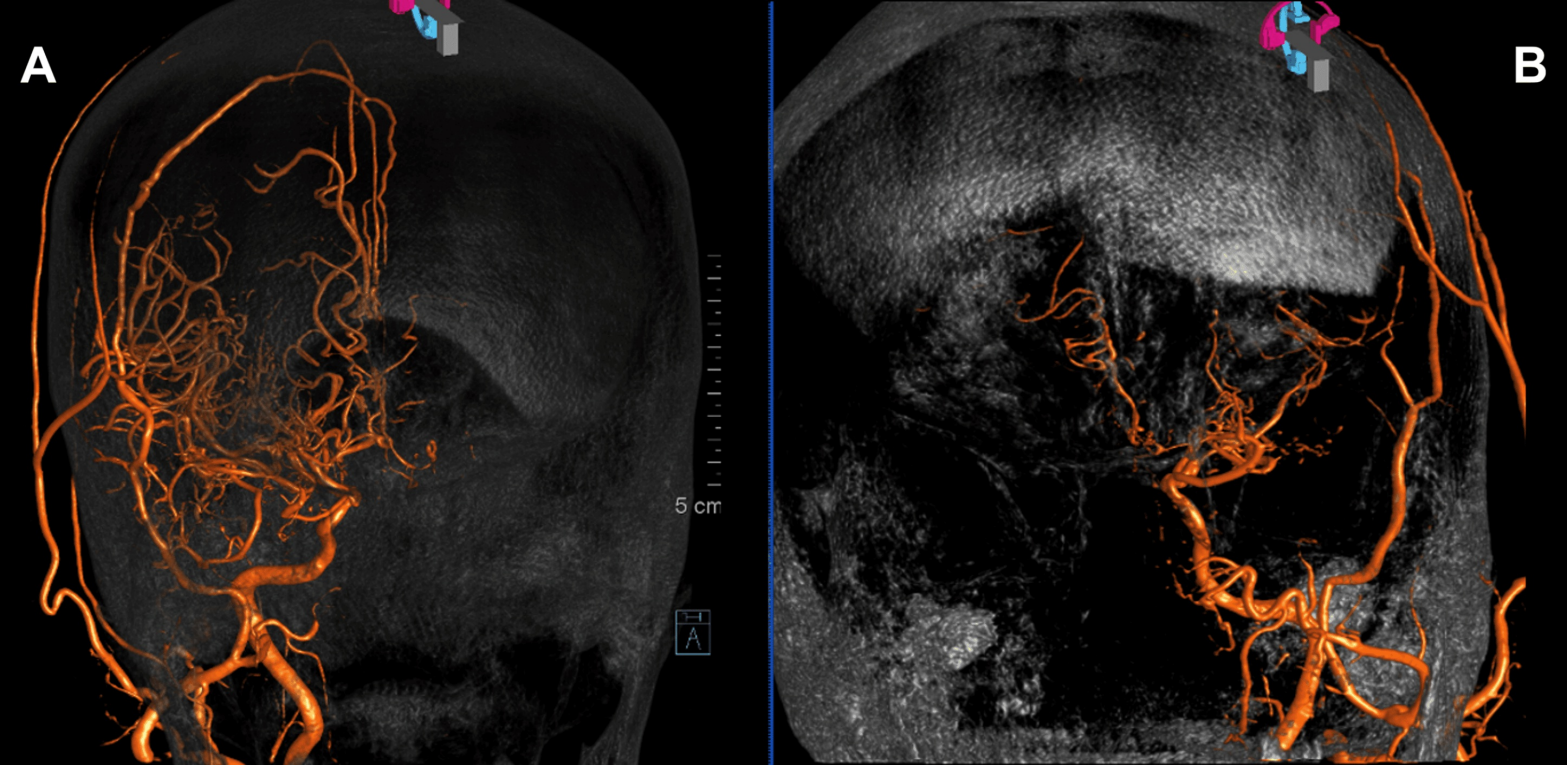
3D volume-rendered images of the angiographic images Left panel: 3D volume-rendered image of the right common carotid artery (RCCA) angiographic run Right panel: 3D volume-rendered image of the left common carotid artery (LCCA) angiographic run

The patient was referred for DSA, which confirmed Suzuki stage 3 moyamoya vasculopathy (Figures 3, 4). Transthoracic echocardiography with bubble study showed an intra-atrial right to left shunt, but no intracranial thrombus. ECG findings were unremarkable and did not contribute to the stroke etiology. However, due to patient age and comorbidities, closure of the intra-atrial defect was not recommended. After careful consideration of risks and benefits, switching to therapeutic anticoagulation was advised by the cardiology service. The patient was discharged to an acute rehabilitation facility following one week of in-hospital physical therapy. At follow-up, the patient demonstrated mild improvement in left-sided weakness and continued participation in rehabilitation therapy.

## Discussion

In this report, we present a rare case of an elderly patient who was incidentally found to have moyamoya vasculopathy during her workup for acute ischemic stroke. The presence of multifocal infarcts in a territory distal from the moyamoya vessels supported multiple coexisting stroke risk factors and influenced the subsequent therapeutic approach. Most commonly, the diagnosis of acute ischemic stroke is based on neurological evaluation and exclusion of other conditions presenting similarly, such as hypoglycemia, Todd’s paralysis, cardiac embolism, and hemorrhagic stroke. This case highlights the role of imaging studies in the diagnosis of acute ischemic stroke and its underlying pathological mechanism. 

In our patient of East Asian descent, the presence of ICA and MCA stenosis, along with a compensatory collateral network of vessels, allowed us to suspect and confirm moyamoya early in the hospital course. Initially, the presenting complaint of acute onset of left-sided weakness was attributed to progressive right MCA stenosis due to moyamoya vasculopathy. Brain MRI, however, demonstrated infarction in a different vascular territory, suggesting an alternative pathophysiologic mechanism and influencing subsequent therapeutic decisions. This pattern of lesion-territory mismatch, where infarct distribution does not correspond to the expected vascular territory of the stenotic vessels, may serve as an important diagnostic clue in distinguishing moyamoya vasculopathy from other stroke etiologies.

While non-contrast CT remains the initial imaging modality in acute stroke, brain MRI provides superior sensitivity for detecting acute ischemia and offers better insight into the underlying pathophysiology [12]. MRI is also capable of identifying stroke mimics, which have been reported in approximately 17% of patients [13], including seizures, migraines, neoplasms, venous infarctions, and posterior reversible encephalopathy syndrome, which would be difficult to rule out by other imaging modalities.

DSA is the gold standard modality for the diagnosis of moyamoya angiopathy [8]. Diagnostic criteria include the presence of stenosis or occlusion of the distal portion of the ICA and its proximal branches, as well as the presence of moyamoya vessels, which are a network of abnormal collateral vessels around the stenotic area. This finding, when idiopathic, is called moyamoya disease. When moyamoya vasculopathy develops secondary to other conditions, it is called moyamoya syndrome [4,8] (Table 2).

**Table 2:** Conditions associated with moyamoya syndrome Table Credits: Ariel Litinski et al. Data compiled from the literature [4,8,15].

Condition
Head irradiation
Meningitis
Autoimmune disorders
Down syndrome
Sickle cell disease
Neurofibromatosis type 1
Brain tumors
Atherosclerosis

The patient had psoriasis, a condition that has been reported in association with moyamoya syndrome. Psoriasis is an independent risk factor for cerebrovascular diseases and diabetes [14]. The incidence of stroke in patients with mild or severe psoriasis is 1/4115 and 1/530 per year, respectively [14]. Although psoriasis has been reported in association with moyamoya syndrome, current evidence remains limited to case reports and small case series. Chen ZY et al. described a series of four patients suffering from psoriasis who eventually developed stroke secondary to moyamoya syndrome [15]. Three out of four patients in his series had intracranial hemorrhage and one TIA. All four patients had active skin psoriasis at the time of presentation [15]. Our patient had elevated inflammatory markers (ESR and CRP) and a positive ANA with a titer of 1:320, which may indicate an ongoing autoimmune process associated with moyamoya syndrome.

The definitive treatment of moyamoya vasculopathy is aimed at the restoration of blood supply to the affected brain areas. Direct surgical approach involves creating connections between the unaffected branch of the external carotid artery (ECA) and cortical arteries, bypassing stenotic segments. Indirect surgical approach implies placement of dura with branches of ECA on the affected cortical area with compromised blood supply, where subsequent ingrowth of the vessels occurs. Medical therapy, consisting of dual antiplatelet therapy, is often used before and after surgical revascularization or when surgery is contraindicated [16].

There is a scarcity of descriptive studies and case reports that have examined moyamoya arteriopathy in the elderly population. In a series of 87 patients with MMD aged 50-67 years old, the authors discovered no significant differences in modified Rankin Scale (mRS) scores between surgically treated patients and conservatively treated patients, with an average Suzuki stage of 4-5 [17]. It is important to note that the presence of diabetes [17] and posterior circulation involvement [17], as well as an advanced age of onset, were identified as predictors of postoperative adverse events. Following a thorough evaluation of the risks and benefits of surgical intervention, a consensus was reached to continue with conservative treatment for our patient with moyamoya vasculopathy.

## Conclusions

Moyamoya disease in elderly patients is rare and may present with atypical clinical and radiographic findings. Acute ischemic stroke in elderly patients with moyamoya vasculopathy presents a diagnostic challenge, as angiographic findings may not correspond to the underlying stroke mechanism. This case underscores the importance of correlating infarct distribution with vascular imaging findings and maintaining a broad differential diagnosis despite the presence of rare cerebrovascular conditions. Recognition of lesion-territory mismatch between angiographic findings and infarct distribution prevented the premature attribution of stroke mechanism to moyamoya and guided an appropriate secondary prevention strategy.
